# A data-driven medication score predicts 10-year mortality among aging adults

**DOI:** 10.1038/s41598-020-72045-z

**Published:** 2020-09-25

**Authors:** Paavo Häppölä, Aki S. Havulinna, Tõnis Tasa, Nina J. Mars, Markus Perola, Mikko Kallela, Lili Milani, Seppo Koskinen, Veikko Salomaa, Benjamin M. Neale, Aarno Palotie, Mark Daly, Samuli Ripatti

**Affiliations:** 1 Institute for Molecular Medicine Finland FIMM, HiLIFE, University of Helsinki, Helsinki, Finland; 2 Finnish Institute for Health and Welfare, Helsinki, Finland; 3 Institute of Computer Science, University of Tartu, Tartu, Estonia; 4 Department of Neurology, Helsinki University Central Hospital, Helsinki, Finland; 5 Estonian Genome Center and Institute of Molecular and Cell Biology, University of Tartu, Tartu, Estonia; 6 Massachusetts General Hospital, Boston, MA USA; 7 Harvard Medical School, Boston, MA USA; 8 Broad Institute of MIT and Harvard, Cambridge, MA USA; 9 Department of Public Health, Faculty of Medicine, Clinicum, University of Helsinki, Helsinki, Finland

**Keywords:** Genomics, Public health, Statistics, Risk factors

## Abstract

Health differences among the elderly and the role of medical treatments are topical issues in aging societies. We demonstrate the use of modern statistical learning methods to develop a data-driven health measure based on 21 years of pharmacy purchase and mortality data of 12,047 aging individuals. The resulting score was validated with 33,616 individuals from two fully independent datasets and it is strongly associated with all-cause mortality (HR 1.18 per point increase in score; 95% CI 1.14–1.22; p = 2.25e−16). When combined with Charlson comorbidity index, individuals with elevated medication score and comorbidity index had over six times higher risk (HR 6.30; 95% CI 3.84–10.3; AUC = 0.802) compared to individuals with a protective score profile. Alone, the medication score performs similarly to the Charlson comorbidity index and is associated with polygenic risk for coronary heart disease and type 2 diabetes.

## Introduction

Health differences among the elderly and the role of medical treatments are topical issues in many aging societies. Older people suffer from multimorbidity^1^, presence of multiple chronic conditions and are susceptible to polypharmacy^2^, use of numerous potentially interacting medications. Both may lead to a severe medication cascade which, in effect, can cause severe adverse drug reactions, decrease quality of life, and even lead to premature death.

Considerable imbalance exists in the number of hospital visits and general medication use among the elderly which manifests in an uneven distribution of health care costs^3^. Understanding these differences better would help target resources and interventions more effectively to those at risk. Increasingly abundant digital health data and modern statistical tools have the potential to facilitate the development of novel ways to measure health differences in the aging population.

Several instruments have been developed to summarize disease diagnoses and exposure to medications into numeric scores using information from hospital databases and medication administration records, e.g. Charlson Comorbidity Index (CI)^4^, Elixhauser Index (EI)^5^, Rx-Risk-V^6^, Medication-Based Disease Burden Index^7^, and Chronic Disease Score (CDS)^8^. These instruments customarily consider a limited number of predefined severe health conditions and consider typically short time windows of a few years, depicting primarily acute changes in health. Given many of these measures are originally derived in hospitalized or institutionalized patient populations and they focus mainly on a priori defined set of severe diseases^9,10^, they could be strengthened by involving long-term prescription medication usage that can capture numerous chronic but less-acute health conditions in the general non-institutionalized population.

The objective of this study is to demonstrate novel data-driven ways to construct health measure from large-scale longitudinal health data and investigate how such score performs and could complement existing classic measures in predicting long-term mortality. In this paper, we derive a new score to measure chronic health differences in an aging population empirically with modern statistical learning methods using 21 years of Finnish pharmacy purchase and death data obtained from high-quality nationwide registries. We demonstrate the applicability of the resulting score in two fully independent prospective cohorts and investigate its relationship to known genetic predictors of late-onset diseases and diagnosis-based comorbidity index.

## Results

We trained 28 medication score candidates (Supplementary Table S1) in the National FINRISK study^11^ where we included a subset of 20,078 participants who were alive and at least 46 years old at the beginning of 2006 (median age 60, IQR 53–67). We followed their mortality 10 years (2006–2015). To train the models with medication data, this mortality was contrasted against purchases 10 years prior to follow-up (1995–2004), with 1 year wash-out in-between (2005). Out of this sample, 15,995 (79.7%) had imputed whole genome genotypes available at the time of the study resulting in 15 million genetic variants per individual.

**Figure 1:**
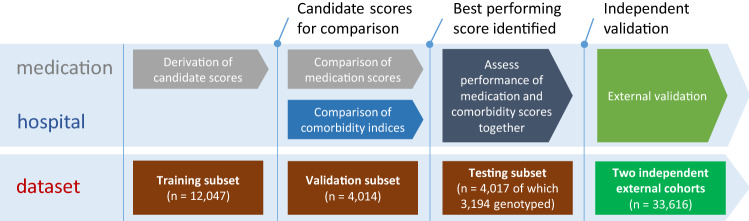
Analysis workflow and FINRISK sample split.

The study was conducted in four consecutive parts as described in Fig. 1. The included participants of the primary cohort (FINRISK) were randomly assigned into three non-overlapping groups: First, we separated a training set to derive candidate medication scores statistically in relation to all-cause mortality (12,047 individuals, 60%). Second, we extracted an independent validation set to compare the predictive performance of alternative medication scores (4,014 individuals, 20%) and two commonly used diagnosis-based comorbidity indices. Third, we evaluated the complement performance of best medication and comorbidity indices together in the testing hold-out set (4,017 individuals, 20%).

Finally, we carried out external validation of the medication scores in two independent cohorts. For Finnish external validation, we used the Health 2000 Survey (H2000)^12^ where we included 5,410 participants who fulfilled the same inclusion criteria as the FINRISK sample. We only had access to the medication data for the H2000, and the cohort was therefore used solely for the validation and comparison of medication scores. Second external validation was conducted in the Estonian Biobank cohort^13^, using data on 28,206 aging individuals.

All included three cohorts were nationwide population studies with no substantial selection or sampling considerations. Cohorts are summarized in the Table 1.

**Table 1:** Description of study cohorts, only aging population.

Name	Data source/owner	Original collection criteria (nationality)	Total included sample size (% women)	Death events in the follow-up period	Age at the beginning of 10-year mortality follow-up	Cohort description	Purpose
National FINRISK study	THL Biobank	Random population sample (FIN)	20,078 (52%)	2,389 (12%)	60 (53–67)	Borodulin et al.^11^	Primary derivation and evaluation set
Health 2000 study	THL Biobank	Random population sample (FIN)	5,410 (55%)	1,082 (20%)	61 (54–72)	Heistaro et al.^12^	External validation (different subset of medications)
Estonian biobank	University of Tartu	Random population sample (EST)	28,206 (68%)	2,517 (8.9%)	61 (53–71)	Leitsalu et al.^13^	External validation (different timeframe)

Data are n (%) or median (IQR).

### Performance of medication scores

A score derived with elastic net regularization and dichotomous medication use indicator (i.e. purchased medicine ever) provided the best overall prediction performance. The result was consistent across all derivation methods: using continuous usage duration or dichotomous “used over 1 year” never improved the performance.

**Figure 2:**
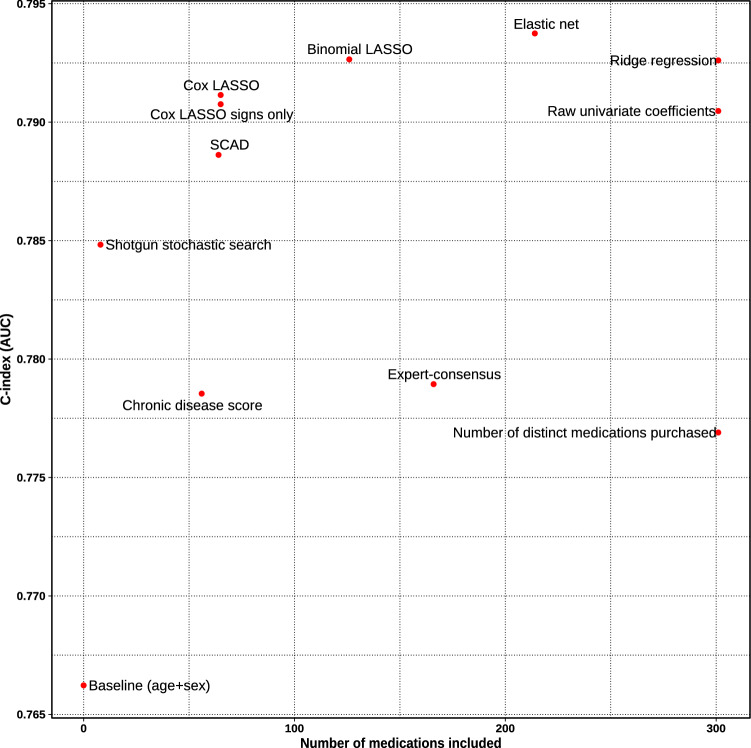
Discriminative performance of the score versus parsimony in the FINRISK validation subset. All based on the “purchased ever” indicator.

A score consisting of 166 medications hand-picked by an expert consensus panel of three medical doctors expectedly predicted mortality better than the simple baseline model (age and gender) alone (C-index 0.766 vs. 0.779, p = 1.83e−7). However, the expert score was inferior to all data-driven statistical approaches including the shotgun stochastic search that included only eight medications (C-index 0.785, p = 0.04) and it performed only marginally better than the classic polypharmacy measure of simply counting the number of distinct medications administered (C-index 0.777, p = 0.06). In our study setup, all statistical learning approaches seemed to outperform the Chronic Disease Score. Scores were built and tested by adjusting for age and sex. Performance of all scores is elaborated in the Supplementary Table S1 and the most interesting scores are illustrated in Fig. 2.

Interestingly, a score involving only non-zero coefficient signs (− 1 and 1) of the Cox LASSO performed nearly as well as the actual Cox LASSO coefficient values (C-index 0.791 for both, p = 0.41, 65 medications). Performance was comparable to binomial LASSO model that included twice as many medications (C-index 0.793, p = 0.14). When comparing it to the best performing elastic net method that employs over 200 medications, the difference in performance seems to be marginal (C-index 0.794, p = 0.05).

After qualitatively considering trade-off between prediction performance, number of medications included, and general parsimony, the Cox LASSO signs-only score was chosen for further analyses. With comparable performance, it considers only a fraction of medications of marginally better performing alternatives. Furthermore, involving coefficient signs only makes the score easily deployable and robust to variance in different data sources and application domains. The resulting integer score is easily interpretable and intuitive without any transformations. The score distribution across the aging population of the whole FINRISK cohort is illustrated in Supplementary Fig. S1 and included medications are listed in Supplementary Table S2. We should note that the shotgun stochastic search score with eight medications could be also an attractive alternative if we preferred extreme parsimony, and it seems to be a direct subset of the 65 medications in the main score.

In order to confirm the result, we took some of the interesting score weights for evaluation to the two external validation cohorts. The results were highly concordant with the FINRISK findings, as summarized in Table 2. Only notable exception is the decreased performance of the elastic net score in the Estonian cohort. It may have an overfitting tendency to Finnish patterns given the large number of medications included. The systematically better absolute performance of all scores in the H2000 cohort can likely be explained with a higher number of mortality events and older age distribution.

**Table 2:** Best performing medication scores evaluated in three separate validation datasets by C-index and Negelkerke R^2^.

Score derivation method	Number of medications included	Main cohort FINRISK validation subset		Replication cohorts			
				R^2^		C-index ∆C baseline	
		R^2^	C-index (95% CI) ∆C baseline (95% CI)	H2000	Estonia	H2000	Estonia
Baseline model (age and sex)	–	0.134	0.766 (0.745–0.788)	0.309	0.098	0.823	0.775
Expert consensus panel	166	0.149	0.779 (0.758–0.800)	0.316	0.107	0.827	0.786
			+ 0.013 (0.006–0.019)			+ 0.004	+ 0.011
Number of medications	301	0.148	0.777 (0.756–0.798)	0.317	0.104	0.828	0.785
			+ 0.011 (0.005–0.017)			+ 0.005	+ 0.010
Shotgun stochastic search	8	0.158	0.785 (0.764–0.806)	0.317	0.102	0.827	0.781
			+ 0.019 (0.01–0.027)			+ 0.004	+ 0.006
Cox LASSO	65	0.164	0.791 (0.771–0.812)	0.324	0.109	0.830	0.793
			+ 0.025 (0.017–0.033)			+ 0.007	+ 0.018
Cox LASSO signs only	65	0.162	0.791 (0.770–0.811)	0.320	0.110	0.829	0.791
			+ 0.025 (0.017–0.032)			+ 0.006	+ 0.016
Ridge regression	301	0.164	0.793 (0.772–0.813)	0.322	0.113	0.830	0.795
			+ 0.026 (0.018–0.034)			+ 0.007	+ 0.020
Elastic net	214	0.166	0.794 (0.773–0.814)	0.324	0.108	0.830	0.784
			+ 0.028 (0.019–0.036)			+ 0.007	+ 0.009

H2000 does not include all the ATC codes of the derivation set.

ATC hierarchies seem to perform comparably when we considered higher level categories, apart from the highest investigated level, therapeutic subgroup, that fared systematically worse (Supplementary Fig. S2). In this study we decided to concentrate on the full ATC code (i.e. chemical substance) as it gives most transparency to the score, is the easiest to apply based on e.g. interview, and the final number of codes included is not substantially higher.

### Performance of comorbidity indices

In addition to medication scores, we compared two commonly used comorbidity indices using inpatient hospital admissions in the validation subset of the FINRISK and all diagnoses in the Estonian Biobank cohort. Although the more complex Elixhauser index has been argued to be superior specifically in hospital settings^14^, Charlson comorbidity index seems to be a better predictor of 10-year mortality in both cohorts (Supplementary Table S3).

### Genetic risk factors

**Figure 3:**
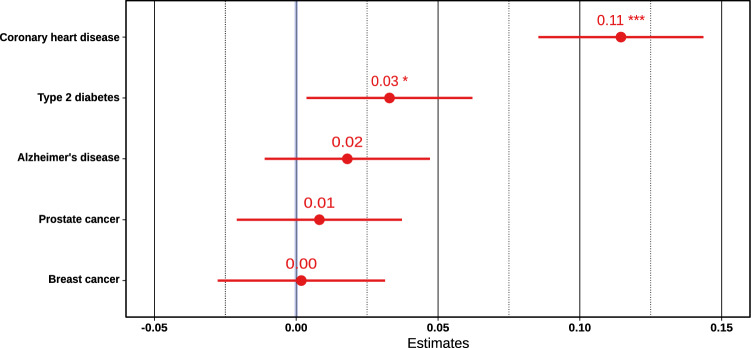
Linear associations of polygenic risk scores with the Cox LASSO signs-only medication score, adjusted with age and sex, within the whole aging population of the FINRISK study (***p < 0.001; **p < 0.01; *p < 0.05).

To investigate the genetic component of the score, we took polygenic risk scores of five major conditions that together account for over 60% of causes of death among the elderly^15,16^. Since genetic risk factors do not cause death directly but through increased disease risk, these polygenic disease risks were combined into a multivariable model to account for any shared genetic etiology and to adjust for age and sex. Together these scores seem to account for only 0.4% of the variance in the score, driven by coronary heart disease and type 2 diabetes (Fig. 3). This is expected given cardiometabolic conditions account for majority of early deaths among the aging and have a strong heritable component.

### Combining medication score, comorbidity index, and genetic risks

When the best medication score and comorbidity index (Cox LASSO signs and CI) were combined additively into one model in the FINRISK hold-out testing subset, measures seemed to complement each other well. Medication score appears to be rather independent of the comorbidity index and adds orthogonal information in relation to the risk of all-cause mortality (Table 3). The CI is only weakly correlated with the medication score (Spearman ρ = 0.233; p < 2.2e−16; Supplementary Fig. S3). Inclusion of five polygenic risks to this model did not seem to increase model performance but neither weakened the estimated effect of the two measures.

**Table 3:** Cox proportional hazards model estimates in the genotyped FINRISK testing subset and Estonian validation cohort in relation to all-cause mortality.

Model				FINRISK (testing subset with genotypes)				Estonian Biobank			
Components	Medications	Comorbidities	Polygenic risk scores	HR_Med_	HR_Comorb_	C-index	p-value (removed variables)	HR_Med_	HR_Comorb_	C-index	p-value (removed variables)
BL						**0.779** (0.757–0.801)				**0.775** (0.767–0.784)	
BL + CI		✓			**1.76** (1.56–1.98)	**0.794** (0.773–0.815)	2.80e−16 (CI)		**1.15** (1.13–1.17)	**0.790** (0.782–0.798)	2.16e−49 (CI)
BL + M	✓			**1.18** (1.14–1.22)		**0.794** (0.773–0.815)	2.25e−16 (M)	**1.19** (1.17–1.22)		**0.791** (0.783–0.799)	3.82e−69 (M)
BL + M + CI	✓	✓		**1.13** (1.08–1.17)	**1.50** (1.31–1.70)	**0.802** (0.781–0.822)	2.49e−8 (M) 3.15e−8 (CI)	**1.15** (1.12–1.17)	**1.09** (1.06–1.11)	**0.796** (0.788–0.804)	1.13e−46 (M) 8.13e−17 (CI)
BL + M + CI + PRS	✓	✓	✓	**1.13** (1.08–1.17)	**1.50** (1.31–1.70)	**0.803** (0.782–0.823)	0.69 (PRS)				

*B* baseline (age and sex covariates), *M* medication score, *CI* Charlson Comorbidity Index, *PRS* polygenic risk scores. Confidence intervals (95%) for estimates provided where applicable.

**Figure 4:**
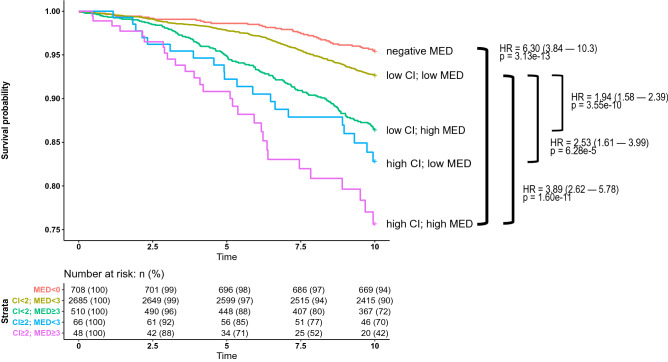
Survival rates in the FINRISK testing dataset and a 10 year follow-up window, stratified by the medication index and Charlson Comorbidity Index. Both based on preceding ten years of medication and hospital data.

We can see a clear difference between mortality rates when we stratify both measures into low and high bins (CI ≥ 2 and MED ≥ 3). Notably, a negative medication score associates with lower risk of mortality compared to individuals with low or zero score (Fig. 4) suggesting a real protective correlation of the negatively weighted medications.

## Discussion

We demonstrated how modern data-driven approaches and longitudinal secondary health data can be used to construct a novel measure of health differences from pharmacy purchases. We tested its association to mortality and inspected relationship to existing comorbidity indices and known genetic risk factors for late-onset diseases. We show that the resulting score is strongly associated with mortality in three datasets independent of the derivation set. When combined with an established comorbidity index, individuals with both elevated medication score and comorbidity index were at over six times higher risk compared to individuals with protective medication profile. The medication score was also associated with genetic risk scores for coronary heart disease and type 2 diabetes.

These findings allow us to draw several conclusions. First, classic indices can be effectively complemented with modern methods by mining large-scale secondary health data such as medication purchase histories. When such data-driven medication score is used alone, it performs similarly as a classic comorbidity index in predicting 10-year mortality in the general aging population. In contrast to most existing comorbidity and polypharmacy measures, our approach poses no presumptions of relevant medication indications and is based purely on an empirical analysis of the register data. Second, now derived medication score could be used as a tool for future population health and genetic research. It can indicate persons among large aging populations in need of a more detailed attention due to their potentially reduced health status and polypharmacy risk. As the score requires no new measurements from the individual and can be applied without complex algorithms on top of existing drug purchase or use databases, it scales effectively to larger populations.

Third, our medication score is a readout of the medication history over the last 10 years. Therefore, the method could be implemented in various health care environments having medication usage records. The resulting score is extremely simple to apply:

1. Identify prescription drugs one has used during the last 10 years (by medical substance, e.g. ibuprofen)
2. Count how many of these drugs are on the left-hand side of Supplementary Table S2 (associated with increased risk). From this number, subtract the count of drugs on the right-hand side of the same table (associated with decreased risk). This gives the medication score where each additional point has a HR 1.18 (95% CI 1.14–1.22).

Based on our replication results, the approach seems to generalize to another health care system and behaves robustly in a cohort where information of some of the included medications is missing.

Built from a predictive perspective, the score associations should not be considered as causal relationships between individual medications and mortality. They rather reflect complex correlations that can be harnessed into a practical measure to indicate aging individuals with an increased risk of long-term mortality and potentially diminished health status. The score associates specifically with life-years gained and does not value the subjective life-quality of prolonged survival in different chronic states. The score is primarily a surrogate for population level health differences, not a substitute for clinical assessment of geriatric frailty or functional impairment.

The methodology was based on fixed-time intervals and did not consider temporal aspects such as changes in medication prescription guidelines or introduction of new medications. The learning method itself could be implemented continuously in any health informatics setting that involves ATC coding and large enough training sample sizes to increase the adaptiveness of the score. In addition, the time windows and wash-out period durations were fixed in our study but could be considered as tunable parameters that could be similarly optimized from the data to maximize prediction power. This could be interesting future research question especially due to potential reverse-causation effect where some medications may end up being prescribed at the point when the lethal disease has already progressed for some time. The learning method itself could be implemented continuously in any health informatics setting that involves ATC coding and large enough training sample sizes to increase the adaptiveness of the score.

We also acknowledge the role of left-truncation and subsequent possible immortal time bias in model estimates. This, however, should not be a major concern when aiming to prediction in similar aging populations and ruling out any profound inferential conclusions about individual medications. Apart from potential left-truncation, the data is derived from nationwide registries that record all deaths, with loss of follow-up occurring only due to moving abroad which we cannot account for. The medication register includes all reimbursed prescription pharmacy purchases and thus excludes medications administered in institutions and over-the-counter medications. Such medications are out of scope of this study whose specific aim was to investigate predictive power of prescription pharmacy purchases in the general non-institutionalized population related to chronic diseases. The Charlson Comorbidity Index was based on standard Quan adaptations of Deyo–Charlson Comorbidity Index and could get marginal benefit from mapping to national Finnish ICD-10 adaptation.

As the aim of the study was to explore empirically the power of modern statistical methods to derive easily applicable measures from a secondary health data, we did not aim to model non-linear medication interactions or individual medication use patterns to the highest detail.

Moreover, our study did not address the role of sociodemographic factors in medication and healthcare usage which could confound some of the results. The Finnish healthcare is strongly based on public funding and medications are publicly subsidized which should mitigate considerably the effect of demographic differences.

## Conclusion

Our study suggests that long-term and large-scale health data can be distilled into a composite measure to infer health differences in the general aging population. Together with Charlson comorbidity index, our novel polypharmacy score identifies 1.2% of elderly population with over six times higher risk of mortality compared to the individuals with a protective medication profile.

Given increasing availability of large-scale health data, statistical learning methods, and abundant computational power, scores aiming for health prediction could be more directly, yet transparently, mined from empirical health data to complement classic measures that are commonly founded on a priori expert opinion. The clinical utility of the newly developed score and relationship to the subjective life quality warrants further studies.

## Materials and methods

To inspect medication usage, morbidity history, and death information in two Finnish cohorts, we used the Register for prescribed medication purchases, the Finnish Hospital Discharge Register, and the National Causes of Death Register^17^. In absence of longitudinal explicit health-related quality of life indicators, we measured health effects as gained life-years by modeling survival. To inspect our endpoint of interest, all-cause mortality, we derived data from the national causes of death register that includes all deaths in the study cohort, timestamps, and relevant diagnosis codes for major, acute, and contributory causes. Validity of the register has been demonstrated by e.g. Rapola^18^.

The Finnish medicine expenses register covers all pharmacy prescription drug purchases since 1995, purchase timestamps, and respective Anatomical Therapeutic Chemical Classification System codes (ATC). The full FINRISK study medication database constituted of 3.4 million medication purchase events in total. Hospital discharge register covers virtually all inpatient hospital visits in Finland since 1969 and has a demonstrated validity for discharges^19^. The register includes a timestamp of hospital stay and relevant diagnoses under the Finnish variant of the International Statistical Classification of Diseases and Related Health Problems scheme (ICD). The full FINRISK hospital discharge register covers nearly 300,000 hospital visits. In this study, we included only hospital visits registered with 10th revision of ICD between years 1996–2015 as ICD-10 was formally taken into use at the beginning of 1996 in Finland.

The Estonian cohort was comprised of participants of the Estonian biobank^13^, and the analyzed data combined time of death from the Estonian Causes of Death Registry and medication prescription information from the Estonian Health Insurance Fund. Diagnosis codes for comorbidity indices were derived from the databases of the Estonian National Health Information System, the Estonian Health Insurance Fund, Tartu University Hospital, North Estonia Medical Centre, and the Estonian Cancer Registry^20^.

In FINRISK, 11.9% of the included individuals deceased within the follow-up period, whereas the number was 20.0% in the H2000 and 8.9% in the Estonian Biobank cohort. The difference can be largely explained by the varying age distributions among the studies.

### Analysis of the time-series register data

**Figure 5:**
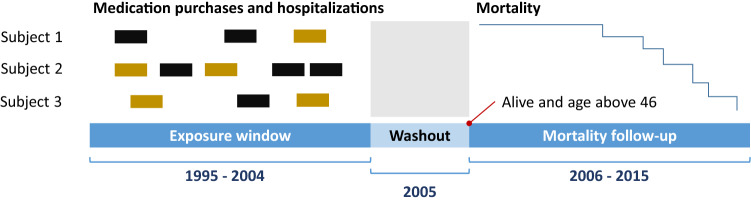
Exposure window and follow-up window illustrated in Finnish cohorts.

In both Finnish cohorts, we split our study time frame identically into three parts (Fig. 5): (i) a 10 year medication purchase and hospital visit exposure window (1995–2004), (ii) a 1-year washout period (2005) to mitigate the effect of palliative care and medications prescribed to terminally ill patients, and (iii) a 10 year follow-up window for all-cause mortality (2006–2015). In the Estonian Biobank cohort, we considered shorter windows: 7 years for exposure (2004–2010) and six years for mortality follow-up (2012–2017) with 1-year washout period in-between (2011).

Longitudinal medication purchases were converted into cross-sectional data points by first generating three alternative indicators for each ATC code: (i) a dichotomous indicator for at least one purchase event within the exposure window, (ii) a dichotomous indicator for at least two purchases 1 year apart, and (iii) a continuous indicator for years between the last and first purchase, a rough proxy for treatment duration. If subject had at least one purchase event for a medication, a generic three-month constant was added to the continuous indicator to account for the duration of the last purchase and to cover single purchase scenarios. Given the ATC classification is a hierarchical coding system, we additionally explored how second, third and fourth levels of codes (i.e. therapeutic, pharmacological, and chemical subgroups) would work with used methods. We did not involve exhaustive register analysis frameworks, as we prioritized parsimony and application simplicity over marginal increases in accuracy.

### Derivation of candidate medication scores and comorbidity indices

Candidate medication scores were derived with high-dimensional multivariable regression methods in the FINRISK training subset as described below. We aimed for a linear additive score that could be calculated effortlessly by taking a weighted sum over different medications. All models were adjusted for age and sex during the training.

First, univariate logistic regressions were run for all individual medications, imposing Firth’s penalty for bias reduction to address separation in rare cases^21^. We used resulting raw univariate regression coefficients as weights for the first score candidate. To find sparse combinations, univariate regression coefficients and medication correlations were also subject to shotgun stochastic search to identify the sparse linear configuration of associated medications with the highest posterior probability^22^.

As a direct multivariable approach, we examined the performance of classical regularized statistical learning methods (L1- and L2-regularization and combination of thereof, i.e. LASSO, ridge regression, and elastic net) and smoothly clipped absolute deviation (SCAD) that has an oracle property of asymptotically finding the true subset of variables under certain assumptions^23,24^. In addition to the above-mentioned methods that all were extensions of a binomial logistic regression, a L1-penalized (LASSO) Cox proportional hazards model was also tested.

As a reference point, we inspected the performance of a conventional score building scheme by including 166 aging-related drugs a priori selected by a consensus panel of three medical doctors from a list of the most commonly used medications in the FINRISK cohort. We further included a common polypharmacy surrogate that simply counts the number of distinct medications used. These two scores imposed equal weights for all included medications.

Two alternative diagnosis-based comorbidity indices evaluated were based on Quan adaptations of Deyo-Charlson Comorbidity Index and Elixhauser-van Walraven Comorbidity Index^25,26^. Comorbidity indices were constructed using the *R-3.5.2* package *icd* and medication scores with *glmnet* and *ncvreg*^27–30^. Chronic disease score was calculated based on methodology of Lix et al.^31^.

### Comparing medication score and comorbidity index performance

Each score candidate was evaluated in the non-overlapping FINRISK validation subset as a continuous variable in a Cox model using death as an endpoint and follow-up time as the time scale. Models were adjusted with age at the beginning of the follow-up and sex. Model fit was compared using Nagelkerke R^2^ and discriminative performance with C-index, a generalized area under the ROC curve measure^32,33^. We also included a reference baseline model that consisted of age and sex only.

Based on numerical validation results and qualitative assessment of score parsimony, we selected the best candidates amidst medication scores and comorbidity indices. These two were then further combined additively into a single Cox model to compare their complementary performance and to evaluate effect estimates in the FINRISK hold-out subsample (testing dataset) together with five genetic risk factors. The proportionality assumption was tested using Schoenfeld residuals and linearity assumption using penalized smoothing splines.

### External validation

Medication score performance was evaluated within the H2000 and Estonian Biobank Cohort to test the robustness and generalizability of our medication score to different follow-up lengths, diverse medication category coverages, and sensitivity to differences in data sources. FINRISK and H2000 cohorts derive their data from the same national register resources and have the same temporal coverage, but medication categories are only partly intersecting (Supplementary Table S4). Estonian cohort, on the other hand, covers virtually all medications sold in Estonia but uses fundamentally different sources for medication information and has a shorter time window. We should also assume that medication prescription patterns differ between these two countries in general.

### Genetic risk factors

To evaluate the relationship of medication score to genetic risk factors, we investigated the association of the best medication score with polygenic risk scores (PRS) based on six million genetic variants. The whole aging genotyped subset of the FINRISK cohort data was used to infer the correlation between best performing medication score and PRS scores. All were pre-adjusted for 10 first principal components and genotyping batch prior to analyses.

### Ethical approval

The study was conducted in accordance with the principles of the Helsinki declaration. Written informed consent was obtained from all the study participants. FINRISK and Health 2000 cohorts were based on study specific consents and later transferred to the THL Biobank after approval by the National Supervisory Authority for Welfare and Health (Valvira). Recruitment protocols followed the biobank protocols approved by Valvira. All participants of the Estonian biobank have signed a broad informed consent that allows follow-up linkage of their electronic health records (EHR), thereby providing a longitudinal collection of phenotypic information for research.

The transfer of the FINRISK and Health 2000 sample collections to the THL biobank has been approved by the Coordinating Ethics Committee of Helsinki University Hospital on 10 October 2014 and by the Ministry of Social Affairs and Health on 9 March 2015. This study was conducted under the THL biobank permissions BB2015_31.1 (FINRISK), BB2017_100 (Health 2000), and the approval of the Research Ethics Committee of the University of Tartu 234/T-12 (Estonian biobank). All DNA samples and data in this study were pseudonymized.

## Supplementary information

Supplementary Information

## Data Availability

The National FINRISK and Health 2000 studies can be applied from the THL Biobank. Access to the Estonian Genome Center Biobank Cohort can be requested from the Institute of Genomics, University of Tartu (https://genomics.ut.ee/en/biobank.ee/data-access). A detailed description of the analytical workflow can be requested from the authors.
