# Supplementary material for: A data-driven medication score predicts 10-year mortality among aging adults
Source: Sci Rep. 2020 Sep 25;10:15760. doi: 10.1038/s41598-020-72045-z (PMC7519677; doi:10.1038/s41598-020-72045-z)

Supplementary Information

A data-driven medication score predicts 10-year mortality among aging adults

Paavo Häppölä, MSc, [1] Aki S. Havulinna, PhD,[1,2] Tõnis Tasa, PhD,[3] Nina Mars, MD, PhD,[1] Markus Perola, MD, PhD,[2] Mikko Kallela, MD, PhD,[4] Lili Milani, PhD,[5] Seppo Koskinen, MD, PhD,[2] Veikko Salomaa, MD, PhD,[2] Benjamin M. Neale, PhD,[6,10,11] Aarno Palotie, MD, PhD,[1,6,7,8,9,10,11] Mark Daly, PhD,[1,6,10,11] Samuli Ripatti, PhD[1,11,12*]

[1] Institute for Molecular Medicine Finland FIMM, HiLIFE, University of Helsinki, Helsinki, Finland.
[2] Finnish Institute for Health and Welfare, Helsinki, Finland.
[3] Institute of Computer Science, University of Tartu, Tartu, Estonia.
[4] Department of Neurology, Helsinki University Central Hospital, Helsinki, Finland.
[5] Estonian Genome Center and Institute of Genomics, University of Tartu, Tartu, Estonia.
[6] Analytic and Translational Genetics Unit, Massachusetts General Hospital, Boston, MA, USA.
[7] Department of Medicine, Massachusetts General Hospital, Boston, MA, USA.
[8] Department of Neurology, Massachusetts General Hospital, Boston, MA, USA.
[9] Department of Psychiatry, Massachusetts General Hospital, Boston, MA, USA.
[10] The Stanley Center for Psychiatric Research, The Broad Institute of MIT and Harvard, Cambridge, MA, USA.
[11] Program in Medical and Population Genetics, The Broad Institute of MIT and Harvard, Cambridge, MA, USA.
[12] Department of Public Health, Clinicum, Faculty of Medicine, University of Helsinki, Helsinki, Finland.

* Corresponding author.

Supplementary Table S1. Medication score comparison in the FINRISK validation subset (* = some models did not converge or coefficient estimate was zero)

Method	Indicator	No. of ATC codes	R2	C-index
**Number of medications**	**Purchased ever (yes/no)**	**301**	**0.148**	**0.777**
Number of medications	Used over one year (yes/no)	301	0.147	0.776
Number of medications	Years of usage (continuous)	301	0.146	0.776
**Expert panel**	**Purchased ever (yes/no)**	**166**	**0.149**	**0.779**
Expert panel	Used over one year (yes/no)	166	0.148	0.778
Expert panel	Years of usage (continuous)	166	0.147	0.776
**Shotgun stochastic search**	**Purchased ever (yes/no)**	**8**	**0.158**	**0.785**
**SCAD**	**Purchased ever (yes/no)**	**64**	**0.160**	**0.789**
SCAD	Used over one year (yes/no)	58	0.158	0.785
SCAD	Years of usage (continuous)	55	0.156	0.784
**Univariate coefficients**	**Purchased ever (yes/no)**	**301**	**0.164**	**0.790**
Univariate coefficients	Used over one year (yes/no)	286*	0.160	0.788
Univariate coefficients	Years of usage (continuous)	301	0.157	0.785
**Cox LASSO (coefficient signs only)**	**Purchased ever (yes/no)**	**65**	**0.162**	**0.791**
Cox LASSO (coefficient signs only)	Used over one year (yes/no)	46	0.158	0.788
Cox LASSO (coefficient signs only)	Years of usage (continuous)	47	0.152	0.782
**Cox LASSO**	**Purchased ever (yes/no)**	**65**	**0.164**	**0.791**
Cox LASSO	Used over one year (yes/no)	46	0.159	0.788
Cox LASSO	Years of usage (continuous)	47	0.156	0.785
**Ridge regression**	**Purchased ever (yes/no)**	**301**	**0.164**	**0.793**
Ridge regression	Used over one year (yes/no)	286*	0.159	0.788
Ridge regression	Years of usage (continuous)	301	0.158	0.787
**Binomial LASSO**	**Purchased ever (yes/no)**	**126**	**0.165**	**0.793**
Binomial LASSO	Used over one year (yes/no)	65	0.162	0.789
Binomial LASSO	Years of usage (continuous)	59	0.157	0.786
**Elastic Net**	**Purchased ever (yes/no)**	**214**	**0.166**	**0.794**
Elastic Net	Used over one year (yes/no)	156	0.162	0.791
Elastic Net	Years of usage (continuous)	82	0.158	0.787
**Chronic Disease Score (CDS)**	**Purchased ever (yes/no)**	**56**	**0.147**	**0.779**

Supplementary Table S2. Lookup table for calculating the medication score. Higher sum indicates increased mortality risk (HR=1.18; 95% CI: 1.14-1.22). (* also picked by shotgun stochastic search)

Associated with increased risk (+1)	Associated with decreased risk (-1)
Acetylsalicylic acid (A01AD05)	Amitriptyline (N06AA09)
Alendronic acid (M05BA04)	Atorvastatin (C10AA05)
Amlodipine (C08CA01)	Betahistine (N07CA01)
Atenolol (C07AB03)	Estradiol (G03CA03) *
Bisoprolol (C07AB07)	Estriol (G03CA04)
Calcitonin (salmon synthetic) (H05BA01)	Fluocortolone (C05AA08)
Carvedilol (C07AG02)	Guar gum (A10BX01)
Citalopram (N06AB04) *	Lorazepam (N05BA06)
Dextropropoxyphene (N02AC04)	Mesterolone (G03BB01)
Diazepam (N05BA01)	Midazolam (N05CD08)
Digoxin (C01AA05)	Mirtazapine (N06AX11)
Dipyridamole (B01AC07)	Nedocromil (R01AC07)
Donepezil (N06DA02)	Norethisterone and estrogen (G03FA01)
Enalapril (C09AA02)	Rosuvastatin (C10AA07)
Fenoterol and ipratropium bromide (R03AL01)	Simvastatin (C10AA01)
Fluticasone (D07AC17)	Terbutaline (R03AC03)
Furosemide (C03CA01) *	Tibolone (G03CX01)
Furosemide and potassium-sparing agents (C03EB01)	Amitriptyline (N06AA09)
Glibenclamide (A10BB01)	
Glimepiride (A10BB12)	
Haloperidol (N05AD01)	
Hydrochlorothiazide and potassium-sparing agents (C03EA01)	
Insulin (human) (A10AB01) *	
Ipratropium bromide (R01AX03)	
Leuprorelin (L02AE02)	
Levodopa and decarboxylase inhibitor (N04BA02)	
Levomepromazine (N05AA02)	
Lisinopril (C09AA03)	
Memantine (N06DX01)	
Metformin (A10BA02)	
Metoprolol (C07AB02)	
Mianserin (N06AX03)	
Nifedipine (C08CA05)	
Oxazepam (N05BA04)	
Perphenazine (N05AB03)	
Platelet aggregation inhibitor combinations excl. heparin (B01AC30)	
Potassium chloride (A12BA01)	
Risperidone (N05AX08) *	
Rivastigmine (N06DA03)	
Salbutamol (R03AC02)	
Tamoxifen (L02BA01)	
Temazepam (N05CD07)	
Theophylline (R03DA04)	
Thioridazine (N05AC02)	
Tiotropium bromide (R03BB04) *	
Tramadol (N02AX02)	
Warfarin (B01AA03) *	
Zopiclone (N05CF01) *	

Supplementary Table S3. Comorbidity index performance in the FINRISK validation subset and Estonian Biobank (C-index).

Comorbidity index	FINRISK validation subset	Estonian Genome Center cohort
Baseline (age + sex)	0.767	0.775
Quan Elixhauser-van Walraven Comorbidity Index (EI)	0.787	0.786
Quan Deyo-Charlson Comorbidity Index (CI)	0.798	0.790

Supplementary Table S4. Intersection of top-level medication ATC categories covered in studied cohorts.

ATC	FINRISK	H2000	ESTONIAN BIOBANK	DESCRIPTION
A08	X		X	Antiobesity preparations, excl. diet products
A10	X	X	X	Drugs used in diabetes
A11	X		X	Vitamins
A12	X		X	Mineral supplements
B01	X		X	Antithrombotic agents
B04	X		X	Serum lipid-reducing agents
C01	X	X	X	Cardiac therapy drugs
C02	X	X	X	Antihypertensives
C03	X	X	X	Diuretics
C04	X	X	X	Peripheral vasodilators
C05	X	X	X	Vasoprotectives
C07	X	X	X	Beta-adrenergic blocking agents
C08	X	X	X	Calcium channel blockers
C09	X	X	X	Agents acting on the renin-angiotensin system
C10	X	X	X	Lipid modifying agents
G02	X		X	Other gynecologicals in atc
G03	X		X	Sex hormones and modulators of the genital system
H02		X	X	Corticosteroids for systemic use
H03		X	X	Thyroid therapy drugs
H05	X		X	Calcium homeostasis
L02	X		X	Endocrine therapy antineoplastic and immunomodulating agents
M05	X		X	Drugs for treatment of bone diseases
N02	X		X	Analgesics
N03		X	X	Antiepileptics
N04	X		X	Anti-parkinson drugs
N05	X	X	X	Psycholeptics
N06	X	X	X	Psychoanaleptics
N07	X		X	Other nervous system drugs
R03	X		X	Drugs for obstructive airway diseases

Supplementary Figure S1. Distribution of LASSO signs medication score in the whole aging subset of the FINRISK cohort. Individual scores range from -6 to 18.

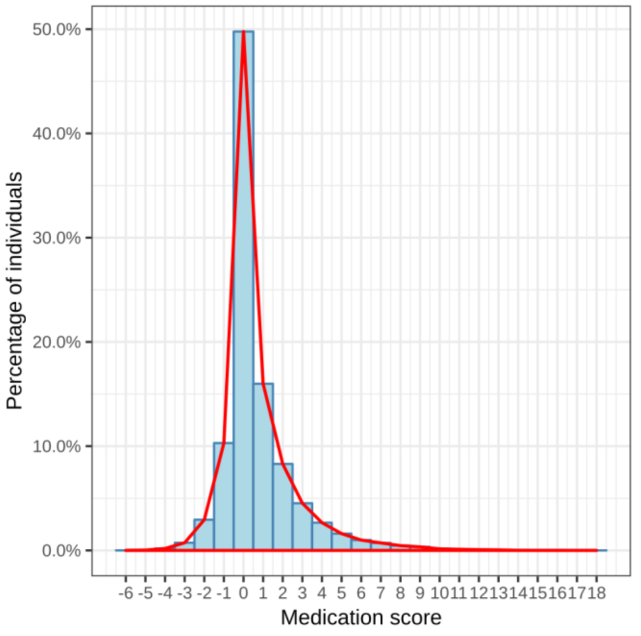

Supplementary Figure S2. Performance of using different ATC hierarchy levels.

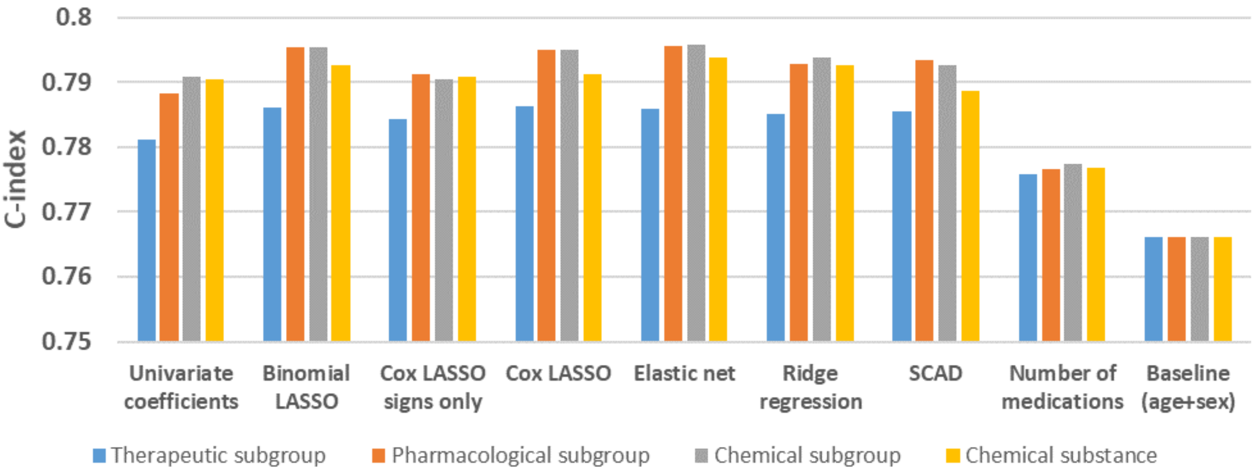

Supplementary Figure S3. Medication score and Charlson comorbidity index in the whole aging population of the FINRISK study. Spearman ρ=0.233; p<2.2e-16. Both are integer scales, points jittered for illustrative purposes.

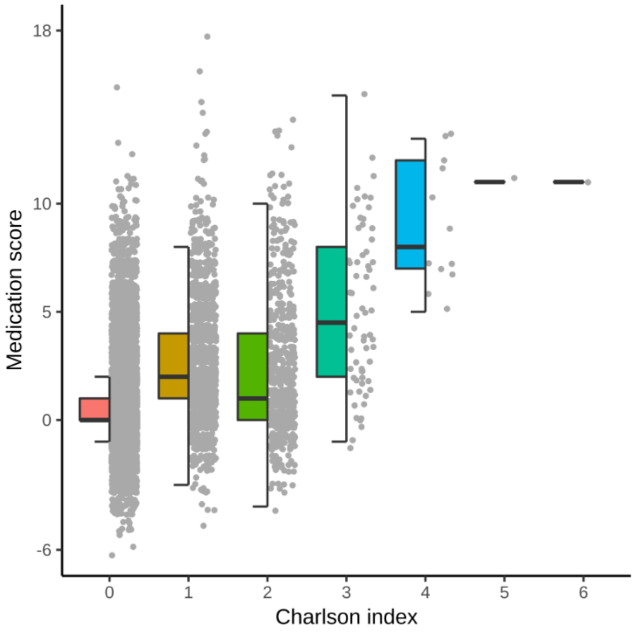

Supplement: Supplementary file 1 — Supplementary Information [file 41598_2020_72045_MOESM1_ESM.pdf]
